# Jing Si Herbal Tea Modulates Macrophage Polarization and Inflammatory Signaling in LPS-Induced Inflammation

**DOI:** 10.7150/ijms.100720

**Published:** 2024-11-11

**Authors:** Meng-Jiun Wei, Kuo-Liang Huang, Hsiu-Fan Kang, Guan-Ting Liu, Chan-Yen Kuo, I-Shiang Tzeng, Po-Chun Hsieh, Chou-Chin Lan

**Affiliations:** 1 Department of Chinese Medicine, Taipei Tzu Chi Hospital, Buddhist Tzu Chi Medical Foundation, New Taipei City, Taiwan.; 2 Division of Pulmonary Medicine, Taipei Tzu Chi Hospital, Buddhist Tzu Chi Medical Foundation, New Taipei City, Taiwan.; 3 School of Medicine, Tzu Chi University, Hualien, Taiwan.; 4 Department of Research, Taipei Tzu Chi Hospital, Buddhist Tzu Chi Medical Foundation, New Taipei City, Taiwan.; 5 School of Chinese Medicine, National Yang Ming Chiao Tung University, Taipei, Taiwan.

**Keywords:** herbal formula, Jing Si Herbal Tea, macrophage polarization, sepsis, systemic inflammatory response

## Abstract

**Background:** Sepsis is a lethal disease due to uncontrolled inflammatory responses. Macrophages play an important role in sepsis-associated inflammation. Jing Si Herbal Tea (JSHT) is a plant-based regimen with anti-inflammatory properties designed to treat respiratory diseases; however, its underlying therapeutic mechanism remains unclear. This study aimed to investigate the effects of JSHT on macrophage polarization and inflammatory signaling in lipopolysaccharide (LPS)-induced inflammation to provide therapeutic approaches for inflammatory diseases.

**Methods:** RAW264.7 cells were stimulated with LPS (1 µg/mL for 16 h) to induce inflammatory responses and treated by JSHT (0.0125% concentration for 4 h) in the experimental groups (Control, JSHT, LPS, Pre-JSHT, and Post-JSHT groups). We investigated the protein and cytokine expression levels using western blotting and enzyme-linked immunosorbent assay (ELISA). Macrophage morphology was observed using immunofluorescence staining. The polarization surface markers were detected by flow cytometry.

**Results:** In the LPS group, the expressions of the inflammatory signaling (pERK, pJNK, and nuclear NFκB) and the pro-inflammatory cytokine levels (TNF-α, IL-1β, and IL-6) were significantly increased, with M1 polarization (CD68+/CD80+) compared to the Control group. In the Pre-JSHT and Post-JSHT groups, the expressions of the inflammatory signaling and the pro-inflammatory cytokine levels were significantly decreased, with a higher M2 polarization ratio (CD163+/CD206+) compared to the LPS group. RAW264.7 cells exhibited filopodia protruding from the cell surface in the LPS group, which were inhibited in the Pre-JSHT and Post-JSHT groups.

**Conclusions:** LPS induced M1 polarization with elevated inflammatory signaling and cytokine levels, while JSHT not only decreased M1 polarization but also promoted M2 polarization with decreased inflammatory responses. We propose JSHT as a potential anti-inflammatory agent against LPS-induced inflammation.

## Introduction

Sepsis, a condition characterized by dysregulated immune responses to bacterial, viral, and fungal infections, claims numerous lives in the world and poses a substantial health challenge worldwide.^1^ Approximately 48.9 million incident cases of sepsis have been documented globally, with 11 million deaths attributed to sepsis.^1^ This life-threatening condition, despite advancements in modern medicine, continues to have a high mortality rate, making it a crucial issue in critical care.^2^ Prolonged stays in intensive care units and hospitals, coupled with the demand for extensive medical interventions, contribute significantly to the overall burden of sepsis on the healthcare system.^2^ Consequently, sepsis not only poses a direct threat to life but also places a substantial strain on healthcare resources. Therefore, investigating the underlying mechanisms of sepsis and developing potential therapies to suppress excessive inflammatory responses in sepsis are critical issues.

Inflammation acts as a defense response against harmful stimuli from microbial pathogens, physical tissue damage, or toxic substances.^3^ On the other hand, hyperinflammation can lead to cytokine storm and damage to local tissues.^3^-^5^ Macrophages function as defensive effector cells against pathogens and play crucial regulatory roles in both innate and adaptive immune responses, including inflammation.^4^, ^6^, ^7^ Macrophages can be divided into two functional subtypes: the classically activated macrophages (M1) and the alternatively activated macrophages (M2).^4^, ^8^ M1-like macrophages, polarized by lipopolysaccharide (LPS), primarily engage in pro-inflammatory responses and produce pro-inflammatory cytokines such as tumor necrosis factor (TNF)-α, interleukin (IL)-1β, and IL-6.^4^, ^8^ In contrast, M2-like macrophages contribute to anti-inflammatory responses and tissue repair by producing anti-inflammatory cytokines such as IL-10 and transforming growth factor beta (TGF-β). 4, 8 Immune system dysregulation due to an imbalance in macrophage polarization has become an increasingly significant concern.^3^, ^8^, ^9^ Addressing inflammation by modulating the balance of macrophage polarization has recently been recognized as a crucial and effective therapeutic strategy.

Jing Si Herbal Tea (JSHT) is an aqueous extract of a plant-based regimen developed by the Buddhist Tzu Chi Medical Foundation to regulate immunity and treat respiratory inflammatory diseases such as COVID-19. 10-12 JSHT comprises various herbal ingredients, including Artemisiae Argyi Folium (*Artemisia argyi*), *Anisomeles indica* (L.) Kuntze, Houttuyniae Herba (*Houttuynia cordata Thunb*.), Perillae Folium (*Perilla frutescens*), Glycyrrhizae Radix et Rhizoma (*Glycyrrhiza glabra*), Platycodonis Radix (*Platycodon grandiflorus*), Ophiopogonis Radix (*Ophiopogon japonicus*), and Chrysanthemi Flos (*Chrysanthemum morifolium*).^10^-^12^ In a recent cohort study involving patients with mild-to-moderate COVID-19, the combination of JSHT with standard management demonstrated improvements in clinical inflammatory markers (C-reactive protein levels) and the severity of pulmonary infiltrate, as evaluated using the Brixia score.^10^ Additionally, a reduced risk of intubation, critical conditions, and mortality has been observed in patients receiving the combined treatment.^10^ Previous studies reported that JSHT shows anti-inflammatory effects.^10^-^13^ However, the mechanisms underlying the therapeutic effects of JSHT are not fully understood.

Since the underlying mechanisms by which JSHT exerts its anti-inflammatory properties have not been fully elucidated, our study aimed to investigate the effects of JSHT on inflammatory signaling pathway and macrophage polarization in LPS-induced inflammation in RAW264.7 cells.

## Materials and Methods

### Reagents and cell Lines

The JSHT (Catalog No.: #4711393151427, a standardized manufactured vacuum-sealed portion) was provided by the Buddhist Tzu Chi Medical Foundation. LPS (#SI-L8274) was purchased from Sigma-Aldrich (St. Louis, MO, USA). The mouse macrophage cell line, RAW264.7, was purchased from Taiwan's Bioresource Collection and Research Center. Dulbecco's Modified Eagle Medium (DMEM; 11965092) and fetal bovine serum (FBS; #A52567-01) were obtained from GIBCO (Thermo Fisher Scientific, Waltham, Massachusetts, USA). Cell Counting Kit-8 (CCK-8; CK04) was purchased from Dojindo Laboratories (Kamimashiki-gun, Kumamoto, Japan). Mammalian cell PE LB (#786-180) was purchased from G-Bioscience (Billerica, MA, USA). Protein assay dye reagent (#500-0006) was obtained from Bio-Rad Laboratories, Inc. (Hercules, California, USA). Polyvinylidene fluoride (PVDF) membranes were purchased from Millipore. The blocking buffer (#37572) was purchased from Thermo Fisher Scientific (Waltham, Massachusetts, USA). Enhanced chemiluminescence was detected using Cytiva software. Nuclear Extraction Kit (#2900) was purchased from Millipore (Billerica, Massachusetts, USA). Penicillin/streptomycin (#CC502-0100) was purchased from GeneDireX (Taipei, Taiwan). DAPI (#D1306) was purchased from Invitrogen (Thermo Fisher Scientific, Waltham, Massachusetts, USA). Paraformaldehyde (#P6148) was purchased from Sigma-Aldrich (St. Louis, MO, USA).

### Antibodies

The primary antibodies for phospho-JNK (pJNK), phospho-ERK 1/2 (pERK), and NFκB were purchased from Cell Signaling Technology (Danvers, Massachusetts, USA). The primary antibody for β-actin was purchased from GeneTex (Irvine, California, USA). Horseradish peroxidase-hybridized secondary antibodies against goat anti-rabbit IgG and goat anti-mouse IgG were obtained from Invitrogen (Thermo Fisher Scientific, Waltham, Massachusetts, USA) and Jackson ImmunoResearch (West Grove, Pennsylvania, USA), respectively. The PE anti-mouse CD163 and FITC anti-mouse CD80 antibodies used for fluorescence staining were obtained from BioLegend (San Diego, California, USA). APC anti-mouse CD68 and Per-CP/Cyaine 5.5 anti-mouse CD206 (MMR) antibodies were obtained from BioLegend.

### Study protocol

The JSHT concentrate was diluted to a final concentration of 0.0125% in DMEM. LPS was prepared in DMEM at a final concentration of 1 μg/mL.^14^ The cells were incubated at 37 °C in a 5% CO_2_ in DMEM enhanced with 10% FBS and 1% penicillin/streptomycin.

**Figure 1:**
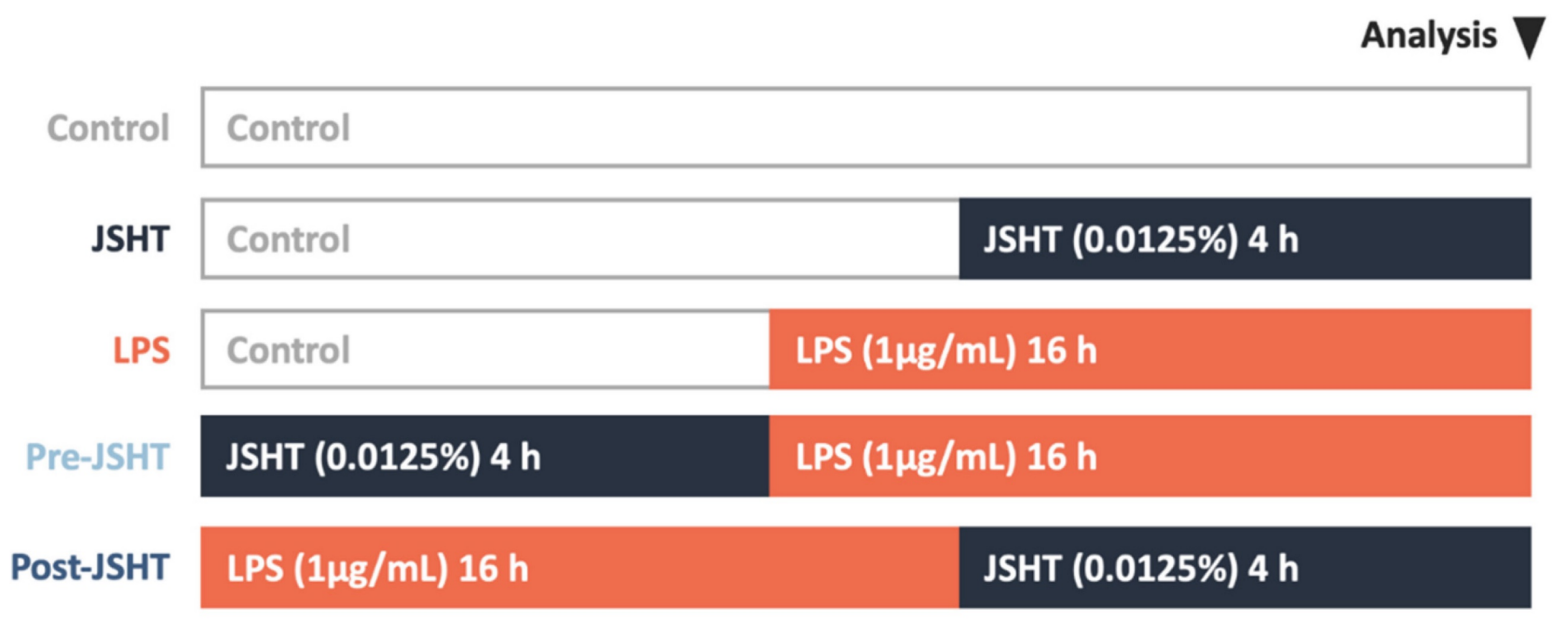
** Study protocol.** Five experimental groups were used in this study: Control, JSHT (0.0125% concentration for 4 h), LPS (1 µg/mL for 16 h), Pre-JSHT (induction of JSHT for 4 h followed by LPS for 16 h), and Post-JSHT (induction of LPS for 16 h followed by JSHT for 4 h) groups.

**Figure 2:**
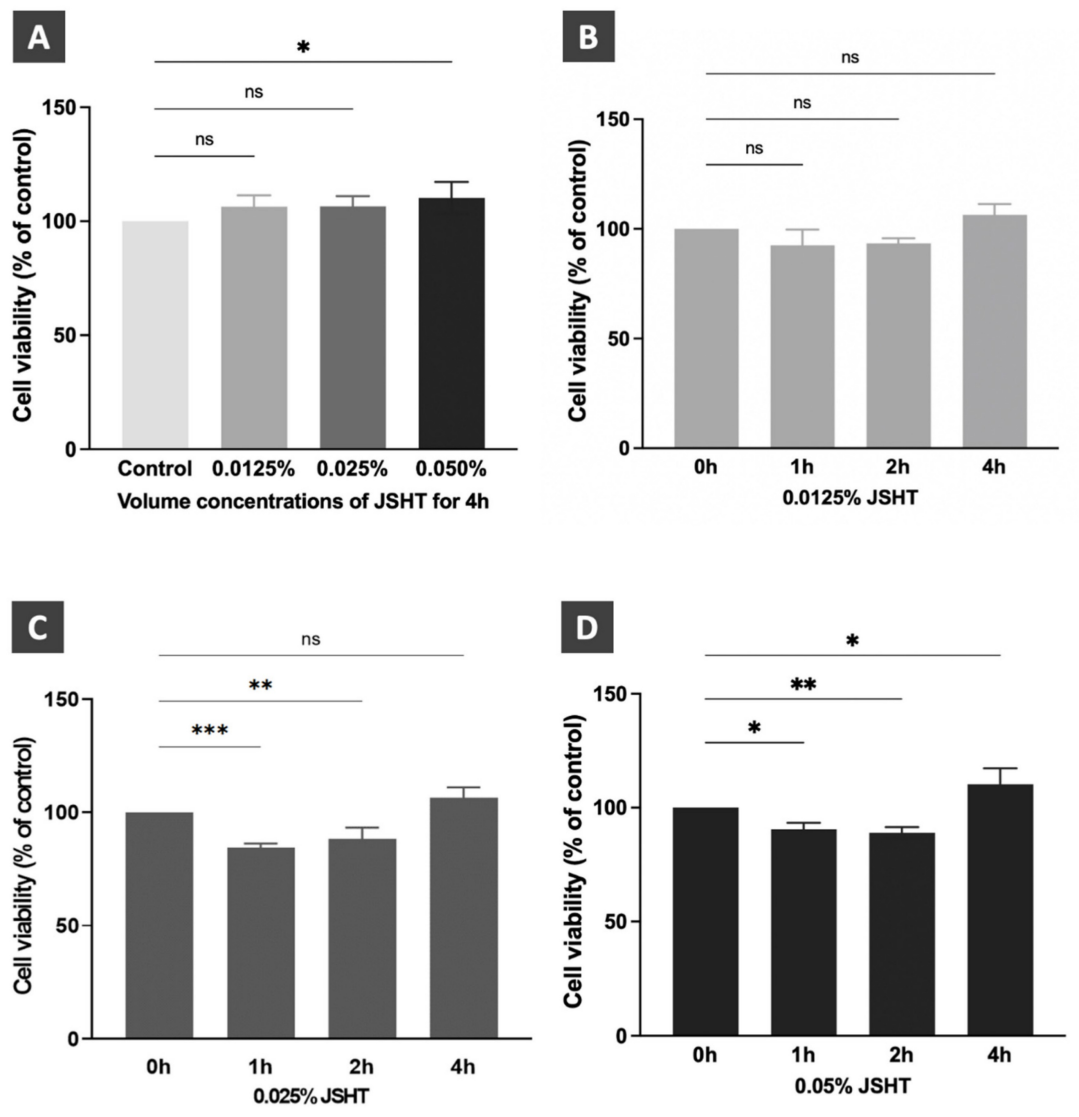
** Dose titration and time course analysis of JSHT in RAW264.7 cells**. (A) Cell viability after JSHT induction at specified concentrations for 4 h. Time course analysis results of JSHT induction at (B) 0.0125% concentration; (C) 0.025% concentration; (D) 0.05% concentration. All data are presented as the mean ± standard deviation (SD). *p < 0.05; **p < 0.01; ***p < 0.001; ns: not significant. The experiment was performed thrice in duplicate.

The study protocol is presented in Figure 1. There were five experimental groups in this study: Control, JSHT (0.0125% concentration for 4 h), LPS (1 µg/mL for 16 h), Pre-JSHT (induction of JSHT for 4 h followed by LPS for 16 h), and Post-JSHT (induction of LPS for 16 h followed by JSHT for 4 h) groups. The experimental conditions were determined according to the dose titration and time-course analysis (Figure 2). After culturing under the conditions in the experimental groups for 20 h, the RAW264.7 cells were harvested, extracted, and subjected to the following experiments.

### Measurement of pERK and pJNK expressions

Whole-cell protein was extracted using Mammalian Cell PE LB, and the protein concentration was quantified using a protein assay dye reagent, according to the manufacturer's protocols. Approximately 50 µg of total protein was applied to each lane and separated by 12% Tris-glycine SDS-PAGE at 140V for 1 h (depending on the molecular weight of the target protein) and then transferred to a PVDF membrane at 200 mA for 2 h.

Primary antibodies were diluted with commercial protein-free blocking buffer according to the manufacturer's instructions. The membrane was soaked for 1h in protein-free blocking buffer, then probed primary antibody at 4℃ overnight. Secondary antibodies were also diluted according to the manufacturer's instructions, hybridized for 1 h at room temperature, and detected using enhanced chemiluminescence via Cytiva software.^15^-^17^

### Measurement of NFκB expression

Nuclear proteins from each experimental group were extracted using a Nuclear Extraction Kit according to the manufacturer's protocol. Well-extracted nuclear proteins were quantified and analyzed by western blotting as described above.^14^

### Morphology of RAW264.7 cells

RAW264.7 were cultured in 6-well plates at a density of 10^4^ cells/well and treated under different conditions, as described in Figure 1. After fixing with 4% formaldehyde for 30 min, RAW264.7 cells were stained with phalloidin for 20-90 min and DAPI for 5 min, and then sealed. The cell morphology was observed using a confocal microscope.^18^

### Measurement of the macrophage polarization surface markers

RAW264.7 cells were treated as described above and harvested. A total of 10^6^ cells were stained with APC anti-mouse CD68, FITC anti-mouse CD80, PE anti-mouse CD163, and Per-CP/Cyaine 5.5 anti-mouse CD206 (MMR) antibodies for 30 min. The cells were then resuspended in 0.5% paraformaldehyde and flow cytometric analysis was performed.^8^, ^9^, ^19^, ^20^

### Enzyme-linked immunosorbent assay (ELISA)

The ELISA kit for mouse IL-1β, IL-6, and TNF-α were purchased from Abclonal (Woburn, Massachusetts, USA), and the ELISA kit for mouse TGF-β and mouse IL-10 were obtained from Invitrogen. The culture medium was collected and the protein concentration was quantified using an ELISA kit according to the manufacturer's protocols.^13^, ^14^, ^16^, ^21^

### Statistical analysis

Statistical analyses were performed using GraphPad Prism 10 for macOS (Version 10.0.1 (170), GraphPad Software, San Diego, CA, USA, www.graphpad.com). Differences between the five groups (Control, JSHT, LPS, Pre-JSHT, and Post-JSHT) were evaluated using analysis of variance (ANOVA). Post-hoc comparisons were performed using the Least Significant Difference (LSD) method to identify specific group differences. Statistical significance was set at *p* < 0.05.

## Results

### Dose titration and time course analysis of JSHT in RAW264.7 cells

We performed dose titration and time-course analyses of JSHT using CCK-8 cell viability assay in RAW264.7 cells.^15^, ^16^ The results are shown in Figure 2. After treatment for 4 h, JSHT at 0.0125% and 0.025% concentrations showed no statistical differences compared to the control condition. JSHT at 0.05% concentration significantly increased the relative cell viability compared to the control condition (*p* < 0.05). Treated with JSHT at 0.0125% concentration for 1, 2, and 4 h showed no statistical differences compared to the control condition (*p* > 0.05; Figure 2B). Treated with 0.025% concentration for 1 and 2 h significantly decreased the relative cell viability (*p* < 0.01), whereas treatment with 0.025% concentration for 4 h showed no statistical differences compared to the control condition (*p* > 0.05; Figure 2C). Treated with JSHT at 0.05% concentration for 1, 2, and 4 h showed statistically significant differences compared to the control condition (*p* < 0.05; Figure 2D). Based on the obtained results, we chose JSHT at a concentration of 0.0125% for 4 h in the subsequent experiments.

### Regulation of pERK, pJNK, and nuclear NFκB expression after LPS and JSHT treatment in RAW264.7 cells

**Figure 3:**
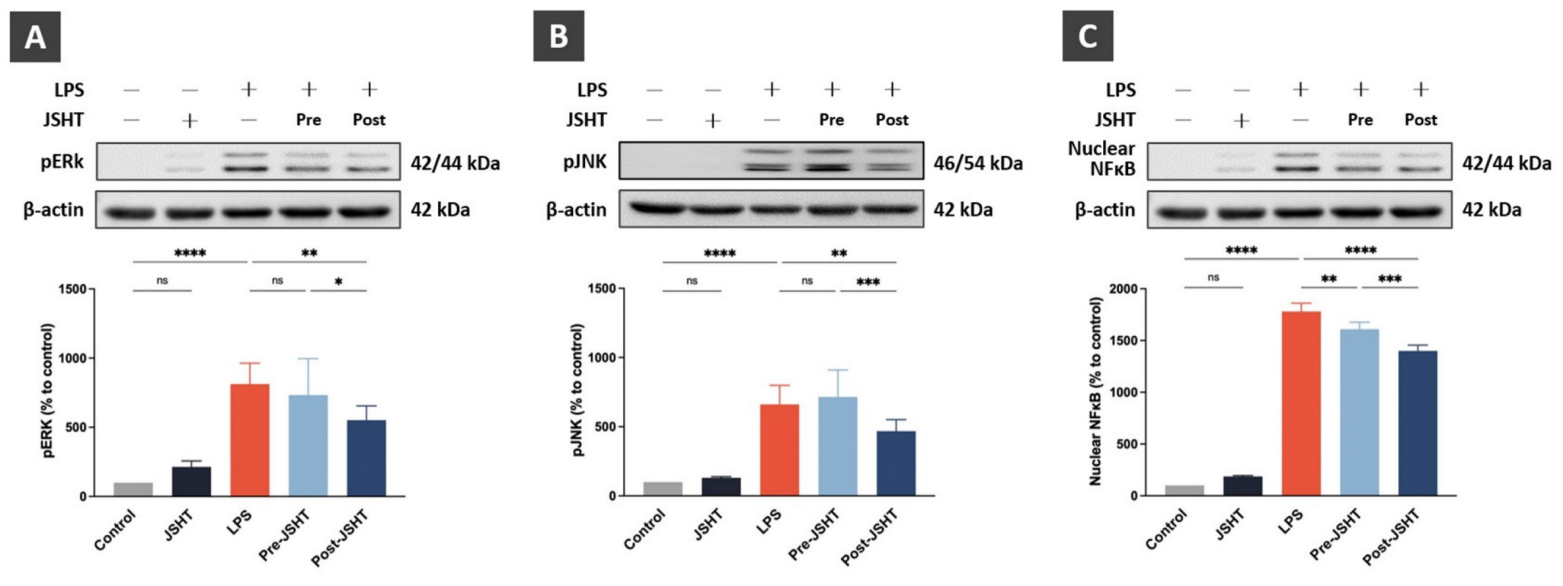
** JSHT inhibited the LPS-induced pro-inflammatory signaling activation in RAW 264.7 cells.** The Western blotting analysis results of (A) pERK; (B) pJNK; and (C) nuclear NFκB. All data are presented as the mean ± SD. *p < 0.05; **p < 0.01; ***p < 0.001; ****p < 0.0001; ns: not significant. The experiment was performed six times in duplicate for pERK and pJNK, and three times in duplicate for nuclear NFκB.

To evaluate the effect of JSHT on the inflammatory signaling pathway in LPS-stimulated RAW264.7 cells, we analyzed the expressions of pERK, pJNK, and nuclear NFκB using western blotting analysis (Figure 3).

Compared to the Control group, the expression of pERK showed no statistical differences in the JSHT group (*p* > 0.05), whereas pERK expression was significantly increased in the LPS group (*p* < 0.0001; Figure 3A). Compared to the LPS group, pERK expression showed no statistical differences in the Pre-JSHT group(*p* > 0.05), whereas pERK expression was significantly decreased in the Post-JSHT group (*p* < 0.01; Figure 3A). The expression of pERK was significantly lower in the Post-JSHT group than in the Pre-JSHT group (*p* < 0.05; Figure 3A).

Compared to the Control group, the expression of pJNK showed no statistical differences in the JSHT group (*p* > 0.05), whereas the expression of pJNK was significantly increased in the LPS group (*p* < 0.0001; Figure 3B). Compared to the LPS group, the expression of pJNK showed no statistical differences in the Pre-JSHT group (*p* > 0.05), whereas the expression of pJNK was significantly decreased in the Post-JSHT group (*p* < 0.01; Figure 3B). The expression of pJNK was significantly lower in the Post-JSHT group than in the Pre-JSHT group (*p* < 0.001; Figure 3B).

Compared to the Control group, the expression of nuclear NFκB showed no statistical differences in the JSHT group (*p* > 0.05), while the expression of nuclear NFκB significantly increased in the LPS group (*p* < 0.0001; Figure 3C). Compared to the LPS group, the expression of nuclear NFκB showed a significant decrease in the Pre-JSHT and Post-JSHT groups (*p* < 0.01; Figure 3C). The expression of nuclear NFκB was significantly lower in the Post-JSHT group compared to the Pre-JSHT group (*p* < 0.001; Figure 3C). These results indicated that JSHT inhibited LPS-induced inflammatory signaling activation in RAW 264.7 cells.

### Morphology alteration of RAW264.7 cells after LPS and JSHT treatment

**Figure 4:**
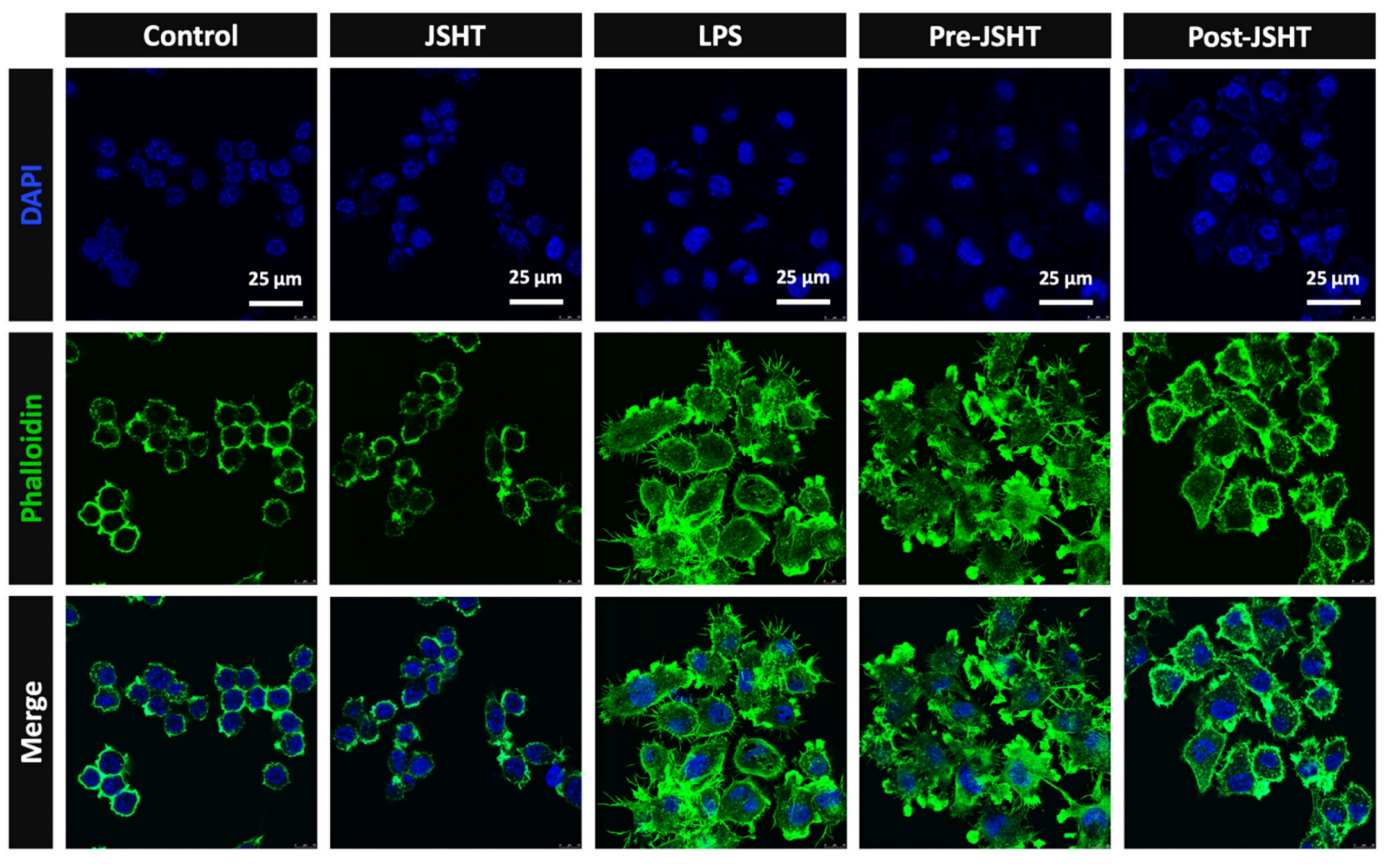
** RAW264.7 morphology alteration in the experimental groups.** Microphotographs of the IF staining results (630X). Blue: nuclei stained with DAPI. Green: cell skeleton and filopodia stained with phalloidin.

To evaluate the morphology of RAW264.7 cells after LPS and JSHT treatment, we performed immunofluorescent staining of phalloidin. The results are presented in Figure 4. The morphology of RAW264.7 cells was circular with smooth surface and few filopodia in the Control group. The morphology of the JSHT group was similar to that of the Control group. In the LPS group, RAW264.7 cells exhibited relatively high cellular volume and obvious filopodia protruding from the cell surface. In the Pre- and Post-JSHT groups, there were morphological alterations with lower cellular volume and fewer filopodia stretching out of the surface compared to the LPS group. The results revealed that JSHT significantly attenuated the LPS-induced morphological alterations in RAW264.7 cells.

### Regulation of macrophage polarization after LPS and JSHT treatment in RAW264.7 cell

**Figure 5:**
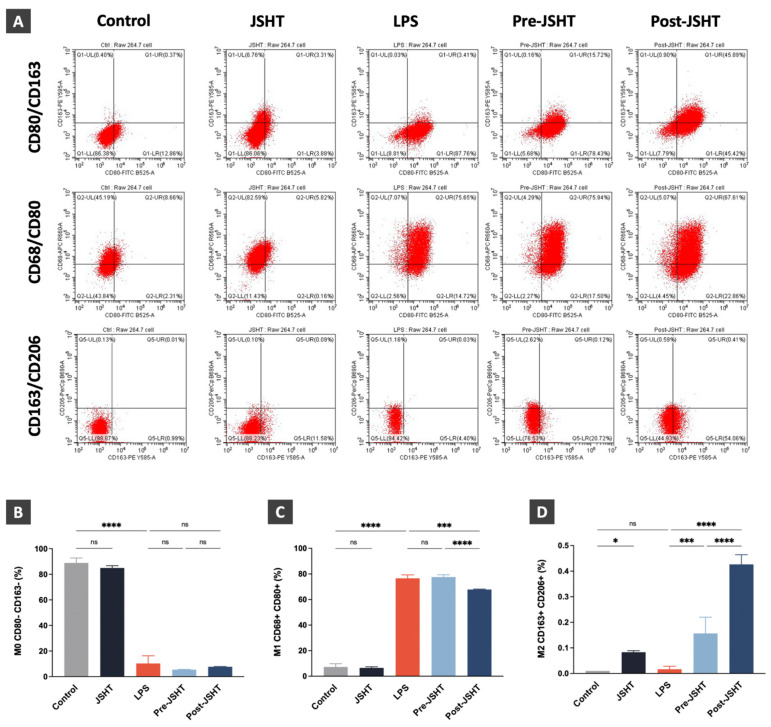
** Expressions of macrophage polarization surface markers in RAW 264.7 cells.** (A) FLOW cytometry analysis results of the expressions of macrophage polarization markers in the RAW 264.7 cells. (B) The expression ratios of M0 macrophage (CD80-/CD163-); (C) The expression ratios of M1 macrophage (CD68+/CD80+); (D) The expression ratios of M2 macrophage (CD163+/CD206+) in the experimental groups. All data are presented as the mean ± SD. *p < 0.05; ***p < 0.001; ****p < 0.0001; ns: not significant. The experiment was performed three times in duplicate.

To investigate LPS- and JSHT- induced macrophage polarization in RAW 264.7 cells, we performed flow cytometry analysis focusing on the expression of macrophage surface markers in the experimental groups. CD68 and CD80 are surface markers of M1 macrophages, while CD163 and CD206 are surface markers of M2 macrophages.^8^, ^19^, ^20^, ^22^ In this study, RAW264.7 cells with CD80- and CD163- markers were identified as M0 macrophage. RAW264.7 cells expressing both CD68+ and CD80+ markers were identified as M1 macrophages. RAW264.7 cells expressing both CD163+ and CD206+ markers were identified as M2 macrophages. The flow cytometry results are presented in Figure 5.

The M0 macrophage expression ratios showed no significant difference in JSHT group (*p* > 0.05) and a significant decrease in the LPS group compared to the Control group (*p* < 0.0001; Figure 5B). There were no significant differences in the Pre-JSHT and Post-JSHT groups compared to the LPS group ((*p* > 0.05; Figure 5B). Regarding the M1 macrophage expression ratios, the results showed no significant change in the JSHT group (*p* > 0.05) and a significant increase in the LPS group compared to the Control group (*p* < 0.0001; Figure 5C). The results showed no significant difference in the Pre-JSHT group (*p* > 0.05) and a significant decrease in the Post-JSHT group compared to the LPS group (*p* < 0.001; Figure 5C). Regarding the M2 macrophage expression ratios, the results showed a significant increase in the JSHT group (*p* < 0.05) and no significant difference in the LPS group compared to the Control group (*p* > 0.05; Figure 5D). There were significant increases in the Pre-JSHT and Post-JSHT groups compared to the LPS group (*p* < 0.001 in Pre-JSHT group and *p* < 0.0001 in Post-JSHT group; Figure 5D). These results revealed that JSHT activated M2 polarization and inhibited LPS-induced M1 polarization.

### Regulation of cytokine expression levels after LPS and JSHT treatment in RAW264.7 cells

**Figure 6:**
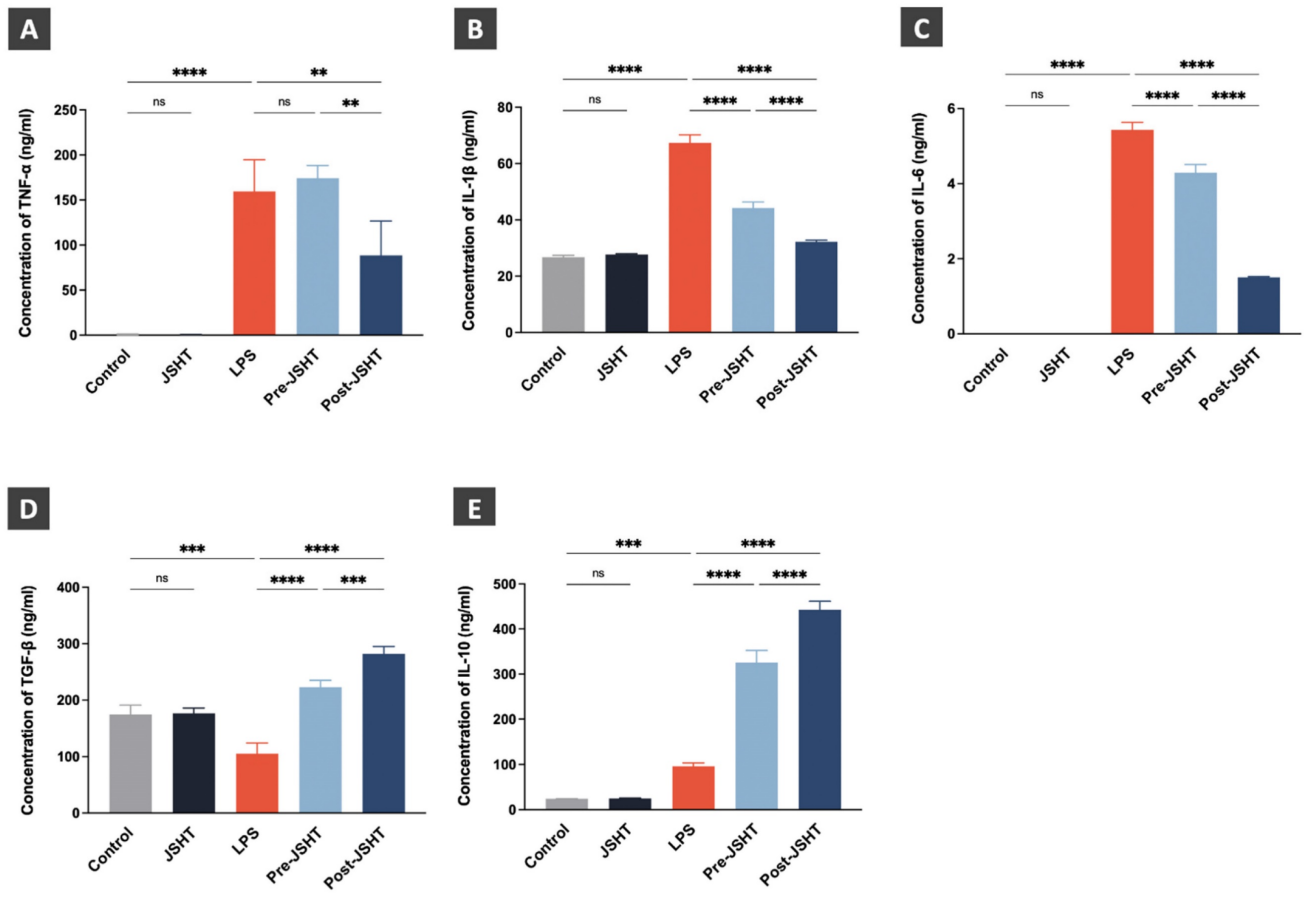
** Cytokine expression levels after LPS and JSHT treatment in the experimental groups.** Concentrations of (A) TNF-α; (B) IL-1β; (C) IL-6; (D) TGF-β; (E) IL-10. All data are presented as the mean ± SD. **p < 0.01; ***p < 0.001; ****p < 0.0001; ns: not significant. The experiments were performed thrice in duplicate.

The effects of LPS and JSHT treatment on cytokine expression levels are presented in Figure 6. The expression levels of the pro-inflammatory cytokines (TNF-α, IL-1β, and IL-6) showed no statistical differences in the JSHT group compared to the Control group (*p* > 0.05; Figure 6A-C). The expression levels of TNF-α, IL-1β, and IL-6 were significantly increased in the LPS group compared to the Control group (*p* < 0.0001; Figure 6A-C). The expression levels of IL-1β and IL-6 were significantly decreased in the Pre-JSHT group compared to the LPS group (*p* < 0.0001; Figure 6B, C). The expression levels of TNF-α, IL-1β, and IL-6 were significantly decreased in the Post-JSHT group compared to the LPS group (*p* < 0.0001; Figure 6B, C). Notably, the expression levels of TNF-α, IL-1β, and IL-6 were significantly lower in the Post-JSHT compared to the Pre-JSHT group (*p* < 0.01; Figure 6A-C). These results indicated that JSHT inhibited LPS-induced inflammation in RAW264.7 cells.

The expression levels of the anti-inflammatory cytokines (TGF-β and IL-10) showed no statistical differences in the JSHT group compared to the Control group (*p* > 0.05; Figure 6D, E). In the LPS group, the expression level of TGF-β were significantly decreased (*p* < 0.001; Figure 6D), and the expression level of IL-10 were significantly increased compared to the Control group (*p* < 0.001; Figure 6E). The expression levels of TGF-β and IL-10 showed a significant increase in the Pre-JSHT group and Post-JSHT groups compared to the LPS group (*p* < 0.0001; Figure 6D, E). Interestingly, the expression levels of TGF-β and IL-10 were significantly higher in the Post-JSHT compared to the Pre-JSHT group (*p* < 0.001; Figure 6D, E). These results revealed that JSHT promoted anti-inflammatory effects during LPS stimulation in RAW264.7 cells.

## Discussion

**Figure 7:**
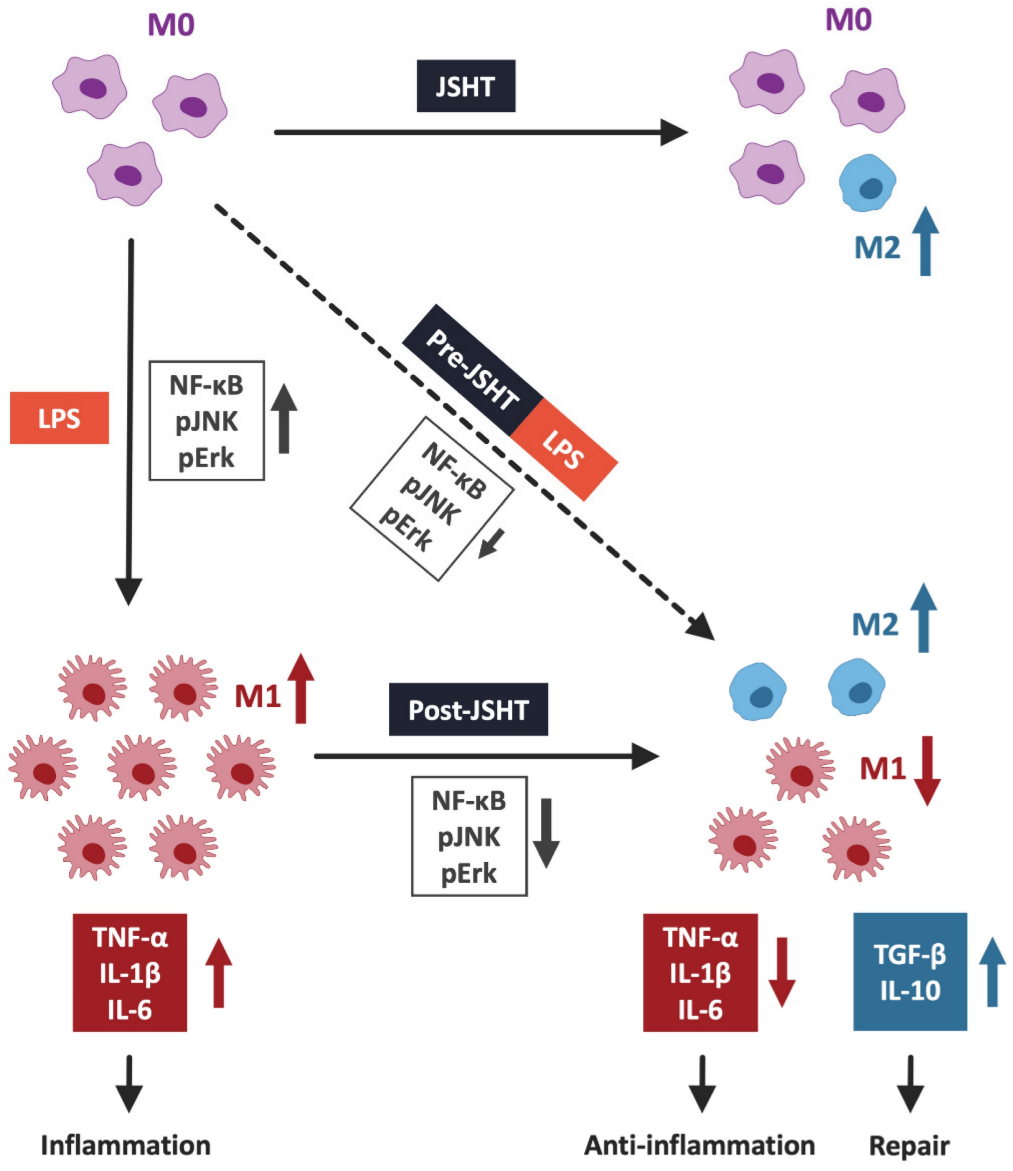
** JSHT alleviated LPS-induced inflammation in RAW 264.7 cells by modulating macrophage polarization.** LPS induction results in the nuclear translocation of NFκB and activates the pERK/pJNK signaling pathway. It promotes macrophages to pro-inflammatory M1 polarization and increases the secretion of pro-inflammatory cytokines, such as TNF-α, IL-1β, and IL-6. JSHT-administration inhibits LPS-induced nuclear translocation of NFκB and downregulates the pERK/pJNK signaling pathway. It raises tendency of macrophages to anti-inflammatory M2 polarization and decreases the LPS-induced secretion of pro-inflammatory cytokines (TNF-α, IL-1β, and IL-6), while increases repair cytokines (TGF-β and IL-10).

This study demonstrated that JSHT inhibited LPS-induced inflammation, in aspects of increased expression of pERK, pJNK, and nuclear NFκB, increased pro-inflammatory cytokine levels (TNF-α, IL-1β, and IL-6), obvious filopodia protrusion, and a higher ratio of M1 macrophages (CD80+/CD68+) in RAW264.7 cells. Additionally, both Pre- and Post-JSHT groups showed an increase in anti-inflammatory cytokines (TGF-β and IL-10) and a higher ratio of M2 macrophages (CD163+/CD206+). Stronger anti-inflammatory effects and M2 polarization were observed in the Post-JSHT group compared to the Pre-JSHT group. The results indicated that JSHT acts as an anti-inflammatory agent against LPS-induced inflammation with respect to macrophage polarization, inflammatory signaling, and pro- and anti-inflammatory cytokine levels (Figure 7).

In this study, we found that LPS-stimulated macrophage polarization to M1, and MAPK signaling pathways (pERK and pJNK), NFκB, and pro-inflammatory cytokines.^23^ LPS serves as the primary structural component of the outer membrane of Gram-negative bacteria and has robust activating effects on macrophages.^24^ The morphological and physiological changes in activated macrophages after LPS stimulation include filopodia protrusion and transformation into M1 macrophages.^9^, ^20^, ^25^, ^26^ During macrophage activation, LPS induced a cascade of events, leading to NFκB nuclear translocation and phosphorylation of ERK and JNK.^27^ Activated NFκB and MAPK pathways up-regulate pro-inflammatory cytokines such as TNF-α, IL-1β, and IL-6.^25^, ^27^ Following stimulation with LPS, macrophages undergo these processes, leading to their polarization into the M1 phenotype.^8^, ^26^

We found that JSHT exerted immune modulatory effects, mitigating the activation of macrophages by dampening the transformation of M1 macrophages and downstream pro-inflammatory responses induced by LPS. JSHT is composed of many components that exhibit anti-inflammatory activity. Houttuyniae Herba has been explored as a potential therapeutic agent because of its anti-inflammatory properties.^10^, ^12^, ^28^, ^29^ It reduces leukocytosis, lowers pro-inflammatory cytokine levels, and downregulates MAPK and NFκB pathways.^28^, ^29^ Perillae Folium effectively inhibits the inflammatory response by deactivating the HMGB1 signaling pathway and significantly suppresses the production of cytokines such as TNF-α and/or IL-6.^30^, ^31^. Glycyrrhizae Radix has anti-inflammatory and immunomodulatory activities, involving NFκB regulation to alleviate inflammatory responses induced by LPS.^32^ *Anisomeles indica* exhibits anti-inflammatory activities, demonstrated by its ability to mitigate NFκB phosphorylation and enhance SIRT1 expression in the ischemia-reperfusion rat model.^33^ In a murine model of LPS-induced acute lung injury, iso-seco-tanapartholide, a compound found in *Artemisia argyi*, inhibited the expression of pro-inflammatory factors TNF-α, IL-1β, and IL-6 induced by LPS.^34^ Platycodi Radix displays anti-inflammatory properties that markedly inhibit the expression of NFκB, TNF-α, IL-6, and caspase-3.^35^ Ophiopogonis Radix displays anti-inflammatory activities, resulting in decreased activation of p65 and phosphorylated IκB in the NFκB pathway, and a substantial reduction in the expression of pro-inflammatory cytokines.^36^-^39^ Chrysanthemi Flos also has anti-inflammatory activity that notably suppresses the NFκB pathways, pro-inflammatory cytokines, and C-reactive protein levels, while mitigating increased leukocytes.^40^-^43^ Luteolin, a natural anti-inflammatory agent, exerts effects against cytokine storms; luteolin treatment significantly reduced the serum levels of pro-inflammatory cytokines, including IL-1β, IL-6, and TNF-α.^42^, ^44^, ^45^ In a recent study, JSHT also demonstrated a protective effect against cytokine-induced injury in normal human lung fibroblasts.^11^

The observed elevation in M2 and related cytokines, TGF-*β* and IL-10 following JSHT administration implies potential anti-inflammatory effects and reparative properties. TGF-β plays a role in repairing substantial DNA damage by facilitating the interaction and localization of repair protein complexes involved in the incision step of nucleotide excision repair.^46^ IL-10 enhances endothelial progenitor cell infiltration and promotes healing after injury.^47^ Few studies have addressed the therapeutic mechanisms of the herbs involved in M2 macrophages polarization and their repair. One report indicated that compounds from *Perilla frutescens* not only inhibited pro-inflammatory cytokines but also enhanced the anti-inflammatory cytokine IL-10.^48^-^51^ Platycodonis Radix inhibits M1 polarization and promotes M2 polarization, leading to an increase in IL-10 and a reduction in TNF-α, IL-6, and IL-1β.^35^, ^52^

### Clinical implication

The clinical implications of the findings in this study on JSHT are significant. This herbal tea was approved by the Ministry of Health and Welfare of Taiwan (registration number MOHW-PM-060 635).^10^-^13^ The ability of JSHT to modulate macrophage polarization and regulate inflammatory responses, particularly in the context of LPS-induced inflammation, suggests its potential therapeutic application in conditions characterized by dysregulated immune responses. The observed inhibition of pro-inflammatory signaling pathways and promotion of anti-inflammatory properties in macrophages following JSHT treatment present promising avenues for interventions aimed at mitigating inflammation-related disorders. Further exploration of JSHT in clinical settings could reveal novel strategies for managing conditions in which macrophage polarization and immune responses play pivotal roles, thereby contributing to the development of effective therapeutic approaches.

### Limitations

This study has several limitations. First, this study was conducted at the cellular level, subsequent investigations involving animals and clinical trials are required to confirm the clinical effects. Secondly, this study focused on the initial phase of LPS-induced inflammation. Further research is imperative to explore the long-term effects of JSHT. Finally, we focused on macrophage polarization, MAPK signaling, and cytokine pathways, which are crucial for inflammation. A more comprehensive exploration of the diverse mechanisms will enhance our understanding of the mechanisms underlying the attenuation of inflammation by JSHT.

## Conclusions

This study highlights the effects of JSHT in modulating macrophage polarization, inhibiting inflammatory responses by suppressing pro-inflammatory cytokines, and enhancing anti-inflammatory cytokines. In the context of LPS-induced inflammation, which is characterized by elevated pERK and pJNK levels, increased pro-inflammatory cytokine levels, and macrophage activation with M1 polarization, while JSHT significantly mitigated these effects. Moreover, JSHT demonstrated positive effects by increasing anti-inflammatory cytokine levels and promoting M2 polarization.

## Abbreviations

IL: interleukin
JSHT: Jing Si Herbal Tea
LPS: lipopolysaccharide
NFκB: nuclear factor kappa-light-chain-enhancer of activated B cells
pERK: phospho-ERK, extracellular signal-regulated kinases
pJNK: phosphor-JNK, c-Jun N-terminal kinase
TGF-β: transforming growth factor
TNF-α: Tumor necrosis factor
